# Mitral Transcatheter Technologies

**DOI:** 10.5041/RMMJ.10115

**Published:** 2013-07-25

**Authors:** Francesco Maisano, Nicola Buzzatti, Maurizio Taramasso, Ottavio Alfieri

**Affiliations:** Cardiac Surgery Department, Ospedale San Raffaele, Milano, Italy

**Keywords:** Mitral regurgitation, mitral valve, percutaneous, valve replacement, valve repair

## Abstract

Mitral valve regurgitation (MR) is often diagnosed in patients with heart failure and is associated with worsening of symptoms and reduced survival. While surgery remains the gold standard treatment in low-risk patients with degenerative MR, in high-risk patients and in those with functional MR, transcatheter procedures are emerging as an alternative therapeutic option. MitraClip^®^ is the device with which the largest clinical experience has been gained to date, as it offers sustained clinical benefit in selected patients. Further to MitraClip implantation, several additional approaches are developing, to better match with the extreme variability of mitral valve disease. Not only repair is evolving, initial steps towards percutaneous mitral valve implantation have already been undertaken, and initial clinical experience has just started.

Mitral valve regurgitation (MR) is the most prevalent valvular heart disease in the community, its prevalence increasing along with population aging and heart failure.^1^ Etiology of MR can be very diverse, and the mechanism of regurgitation is variable according to the underlying anatomo-functional lesions. Organic lesions are most commonly secondary to degeneration of connective tissue with localized or diffuse alterations of the annulus, leaflets, and chordae, leading to prolapsing lesions and annular dilatation. Beyond degenerative MR (DMR), organic MR can be of post-inflammatory, infective etiology, or associated to other rare diseases. In contrast, functional MR (FMR) is characterized by absence of structural lesions, and mitral insufficiency is due to sub-valvular and valvular deformations caused by left ventricular remodeling and dysfunction.^2^ The natural history of severe MR is unfavorable, leading to left ventricular (LV) failure, atrial fibrillation, stroke, and death.^3^ Conventional treatment of significant MR is surgery, either repair or replacement. This is particularly true for DMR. Surgery for DMR is very safe and effective, and, in relatively young patients with few co-morbidities, hospital mortality is below 1%.^4^ As a consequence, the current approach is to perform early surgery with mitral valve reconstruction to guarantee preservation of life expectancy and quality of life similar to a comparable healthy population.^5^ On the other hand, the landscape of FMR therapies is wide and full of controversies. Functional MR is dependent on loading conditions, and timing of surgery can be difficult to establish, particularly when patients are evaluated under aggressive therapy and in resting conditions.^6^ Surgery for FMR carries higher risk compared to DMR, and its prognostic value as well as the best surgical treatment for functional MR is still debated.^7^,^8^ As an alternative to surgery, FMR can be managed with medical therapy or other therapies acting on left ventricular function including resynchronization.

The Euro Heart Survey data^9^ revealed that up to 50% of symptomatic patients hospitalized with severe MR are not referred for surgery, mainly due to advanced age (>70 years), co-morbidities, and depressed LV function, so that the surgical risk is considered too high. In the subgroup of patients aged 80 years and older, surgical treatment was performed only in 15%, as compared to 60% in patients aged 70 years and younger. Data from the Society of Thoracic Surgeons (STS) database confirm that surgical risk increases with age, and it is higher for replacement and in combined procedures involving coronary artery bypass grafting.^10^,^11^ Badhwar et al. have recently utilized a linkage between the STS database and the longitudinal claims data from the United States Medicaid and Medicare Services to investigate the long-term durability and outcomes after mitral valve repair in 14,604 patients older than 65 years between 1991 and 2007.^10^ Approximately 53% had symptoms of severe heart failure at the time of operation (NYHA class III–IV). Overall, operative mortality was about 2.6%. By age quartile of 65–69 years, 70–74 years, 75–79 years, and 80 years or greater, the operative mortality was 1.7% (72 of 4,311), 1.9% (85 of 4,426), 3.4% (126 of 3,669), and 4.3% (95 of 2,198), respectively. Operative mortality was significantly higher among patients with advanced heart failure at the time of operation (1.5% in NYHA class I or II versus 3.3% in NYHA class III or IV, *P* < 0.0001). Mean follow-up was 6 years. The 10-year survival rate after mitral valve repair was 57%, identical to that of the normal age- and sex-matched US population. Five-year survival was 68% among patients with NYHA class III–IV compared with 85% among those with NYHA class I–II (hazard ratio class III–IV versus class I–II: 2.65). The numbers of observed events for mitral reoperation, heart failure, bleeding, and stroke were 552 of 14,604 (3.7%), 2,681 of 14,604 (18.4%), 1,051 of 14,604 (7.2%), and 1,131 of 14,604 (7.7%), respectively. Advanced preoperative symptoms were strongly associated with 5-year readmission for congestive heart failure after successful mitral valve repair (NYHA class IV 33% versus NYHA class I–II 14%; hazard ratio 2.76).

Seeburger et al. recently reported the single-center experience with 2,053 elderly (defined as 70 years or older) patients who underwent mitral valve (MV) surgical procedures with or without associated procedures.^12^ Seventy-seven patients (3.1%) died within 30 days after the operation. Postoperative low cardiac output syndrome was seen in 316 patients (12.6%) and treated with inotropic support, the application of an intra-aortic balloon pump, or both. Cerebrovascular accidents, including transient and persistent neurologic deficits, occurred in 105 patients (4.2%). Implantation of a pacemaker during the postoperative course was indicated in 268 patients (10.7%). Incidence of acute renal failure was 16.7% (418 patients). Patients were discharged from hospital for further rehabilitation treatment at 17.3 ± 11.7 days after operation. Concomitant coronary artery bypass surgery (CABG) was a significant risk factor for increased early mortality (odds ratio 2.3, *P* = 0.016). Age stratification revealed a significantly better 5-year survival for patients between the ages of 70 and 75 years of 58.6%, compared with 52.9% at the age of 75 to 80 years, and 47.9% at the age of >80 years. Associated co-morbidities (including diabetes, pulmonary disease, perioperative hemodialysis, low ejection fraction, and need for associated tricuspid valve procedure) were associated with an increased risk of late death.

These data support the evidence that comorbidities are the real burden in the successful treatment of elderly patients undergoing MV procedures. In this setting a number of minimally invasive transcatheter techniques are emerging to treat MR in high-risk and elderly patients, as an alternative to conventional surgery. Mitral transcatheter interventions carry the hope of minimizing risks while preserving clinical efficacy of surgical repair and replacement. As such, transcatheter interventions may improve outcomes by reducing risks in elderly patients, with reduced left ventricular function or with co-morbidities, and could open the way for earlier interventions, particularly in the field of FMR.^13^

Multiple technologies and diversified approaches are under development. They can be categorized based on the anatomical and pathophysiological addressed target.

## LEAFLET PROCEDURES

All these procedures act directly at the leaflet level with the final goal of improving leaflet coaptation and reduce the effective regurgitant orifice.

**Figure 1:**
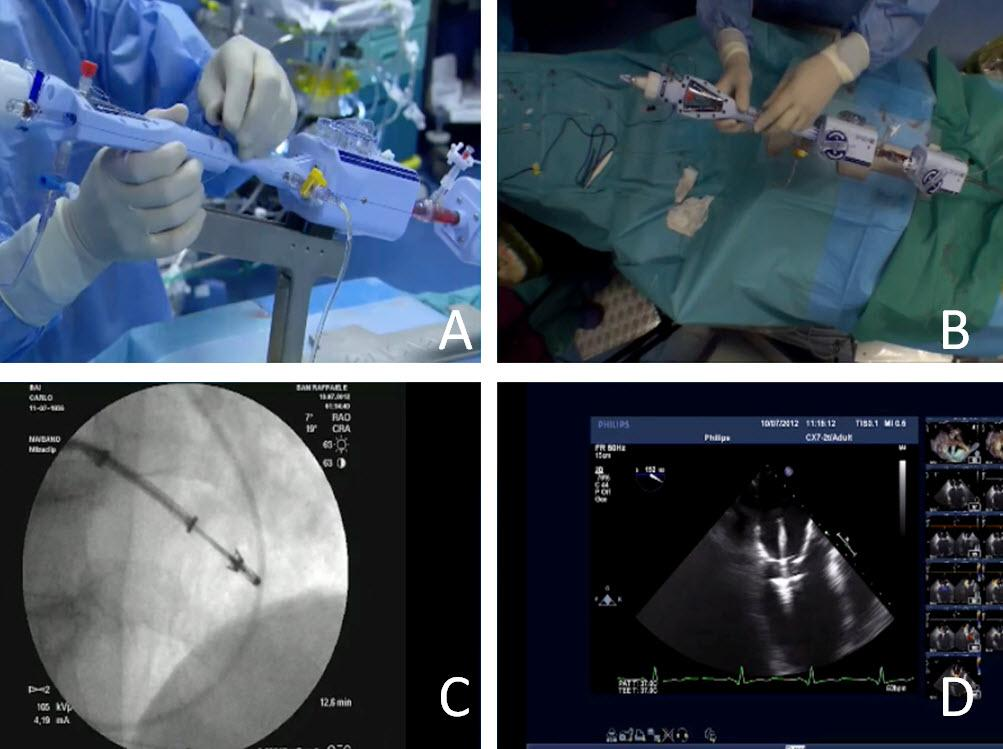
**MitraClip^®^ System.** **A** and **B**: The delivery system in use from lateral and above views, in a live case. **C:** Fluoroscopic view. **D:** Echo-view during grasping.

The most advanced technology under this category is the *MitraClip*^®^*System* (Abbott Vascular, Inc., Menlo Park, CA, USA). It is the most widely used transcatheter mitral device (more than 8,000 procedures worldwide). The MitraClip system was almost directly derived from the surgical edge-to-edge technique^14^,^15^ that corrects MR by suturing the leaflet edges at the site of regurgitation, regardless of the underlying mechanism of dysfunction. MitraClip is effective to treat both degenerative MR (DMR) and functional MR (FMR). The MitraClip system consists of two parts: the clip delivery system and the steerable guide catheter. The clip delivery system consists of three major components: the delivery catheter, the steerable sleeve, and the MitraClip device. The clip delivery system is introduced into the body through a steerable guide catheter, which includes a dilator. The clip delivery system is used to advance and manipulate the implantable MitraClip device for proper positioning and placement on the mitral valve leaflets. The system is designed to deploy the implant in a way that requires multiple steps to ensure safe delivery of the device. The MitraClip device is a single sized, percutaneously implanted mechanical clip (Figure 1). The MitraClip device grasps and joins the mitral valve leaflets resulting in fixed approximation of the mitral leaflets throughout the cardiac cycle. The MitraClip device is placed without the need for arresting the heart or cardiopulmonary bypass. The implantable MitraClip device is fabricated with metal alloys and covered by polyester. The MitraClip device arms can be adjusted to any position from fully opened, fully inverted, and fully closed. These positions are designed to allow the MitraClip device to grasp and approximate the leaflets of the mitral valve using the controls on the delivery catheter handle. The MitraClip device can be locked and unlocked and repeatedly opened and closed. The gripper can be raised or lowered repeatedly. The procedure is performed in the cardiac catheterization laboratory with echocardiographic and fluoroscopic guidance while the patient is under general anesthesia. To access the left heart, standard transseptal catheterization is performed, and the guide catheter is then percutaneously inserted into the femoral vein. The delivery catheter is inserted into the guide, and the clip is positioned above the mitral valve. Manipulation of the steering mechanism on the handles of the guide and delivery catheter positions the clip on the mitral valve. The clip is actuated (i.e. opened and closed, locked, deployed) through manipulation of levers on the handle of the delivery catheter. More than one clip can be delivered, and each one remains repositionable until detachment.

The first MitraClip procedure was performed about 10 years ago.^16^ Subsequently, one randomized trial has proved MitraClip safety^17^ and short- to mid-term efficacy in selected patients.^18^ In the so-called “real world” the MitraClip therapy is usually reserved to high-risk and extreme patients (mainly due to age, co-morbidities, and left ventricle dysfunction). Despite this, it has confirmed an excellent safety profile (30-day mortality 2%–5%) and acceptable mid-term outcomes (1-year survival 75%–85%, 1-year freedom from MR >2+ 80%) especially in terms of improvements in symptoms and quality of life.^19^–^22^ Major advantages of the MitraClip are its excellent safety even in end-stage patients and the possibility to operate on the beating heart, monitoring the efficacy of the implant during the procedure. On the other hand, MR recurrence (higher than in the surgical experience) is the most debated issue. Longer follow-up is needed to verify MitraClip outcomes in terms of MR recurrence and clinical benefit (survival and quality of life).

European guidelines assigned an indication class IIb, level of evidence C, signifying that MitraClip may be considered in patients with symptomatic severe MR despite optimal medical therapy, who are judged inoperable or at high surgical risk by a heart-team, and with life expectancy greater than 1 year.^23^ The randomized RESHAPE and COAPT trials, respectively in Europe and the US, are currently evaluating the benefit of MitraClip compared to optimal medical therapy to support a higher recommendation class in the forthcoming guidelines.

A different approach to obtain transcatheter leaflet repair is off-pump adjustable chordal implantation, for which several devices are currently under development.

The *Babic device* (from the name of the inventor, Uros Babic, MD)^24^ creates two continuous guiding tracks from the left ventricular puncture site through the target leaflet. The device is then exteriorized via the transseptal catheter and femoral vein. A polymer loop is apposed onto the venously exteriorized guiding tracks via docking adapters and is anchored onto the atrial leaflet surface by retracting the guiding tracks from the epicardial end. An elastic polymer tube is interposed between the leaflet and the free myocardial wall and secured to the epicardial surface by an adjustable knot.

The *MitraFlex* (TransCardiac Therapeutics, Atlanta, GA, USA) places an anchor in the inner LV myocardium and another on the leaflet via a thoracoscopic transapical approach and connects the two with a synthetic chord; it can also perform an edge-to-edge repair at the same time.

The *NeoChord* (NeoChord, Inc., Minnetonka, USA)^25^ uses a mini-thoracotomy transapical access to capture the leaflet, and the chords are then exteriorized, adjusted, and tightened to the left ventricular myocardium.

**Figure 2:**
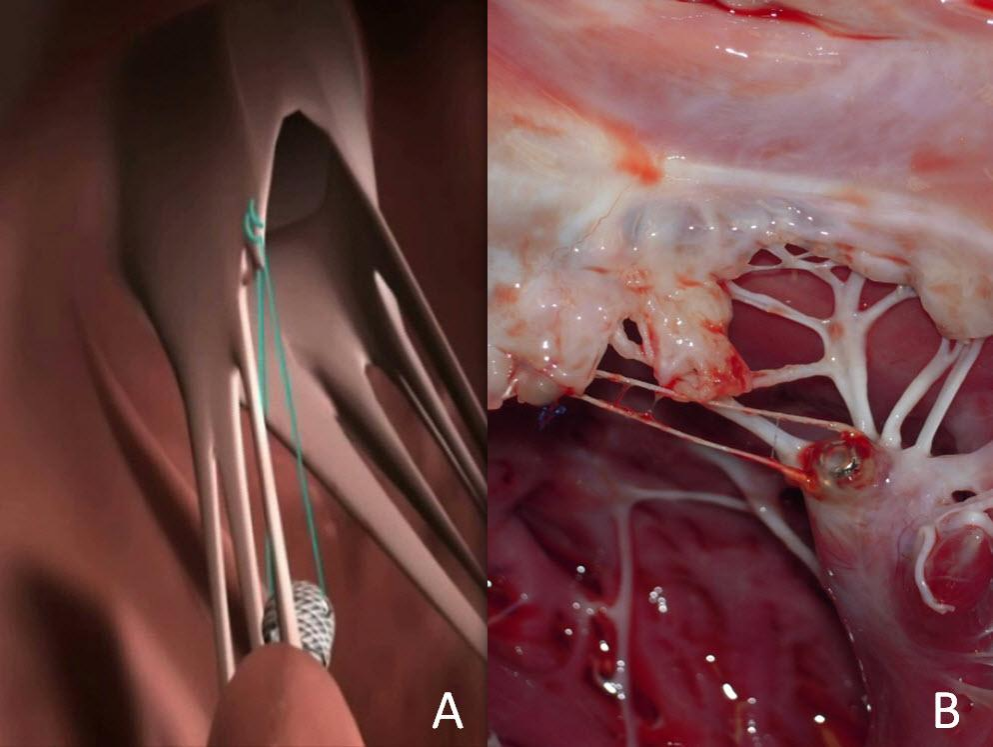
**The V-Chordal Device, a Surgically Implantable Adjustable Chordae Device.** The device can be implanted with a sutureless approach, which opens the perspective of fully percutaneous implants. The length of the chordae can be adjusted on the beating heart for optimal coaptation. **A:** A drawing depicting the concept. **B:** An animal model post-mortem examination showing the adjusting device in the tip of the papillary muscle with chordae attached on the free edge of the anterior leaflet.

The *V-Chordal* (Valtech Cardio Ltd, Or-Yehuda, Israel)^26^ system uses a slow rotation of a helical element to fixate the chorda to the papillary muscle (Figure 2). Currently, the chordae are then sutured to the leaflets by direct vision through a mini-thoracotomy left atriotomy approach, but a clip-attaching mechanism is already under development, to allow transfemoral access.

Alternative techniques for leaflet repair have been proposed. The *Percu-Pro* (Cardiosolutions, Stoughton, USA) is a space-occupying “buoy” anchored at the LV apex through a transseptal approach that should fill the gap between leaflets. The *Thermocool* irrigation ablation electrode (Biosense Webster, Inc., Diamond Bar, CA, USA) delivers, in the context of degenerative disease, radiofrequency energy to the leaflets to provoke shrinking and reduced motion.^27^

## ANNULOPLASTY PROCEDURES

Annuloplasty is a fundamental step to achieve effective and durable results after surgery.^28^ To achieve similar outcomes, transcatheter procedures should probably incorporate annular remodeling. In addition, the lack of a reliable annuloplasty device is impacting the eligibility for transcatheter interventions. Up to a third of patients screened for MitraClip are refused due to unfavorable anatomy, including annular dilatation.^29^ Transcatheter annuloplasty may therefore both improve outcomes and expand therapeutic indications. Different devices to reduce and reshape the mitral annulus are at different stages of research and development, addressing different anatomical and pathophysiological concepts.

### Indirect Annuloplasty (Sinoplasty)

The coronary sinus (CS) encircles the posterior mitral annulus, and it may allow devices to be delivered to affect indirectly the posterior mitral annulus geometry. Although the CS approach is a consolidated procedure, it has important limitations: the CS is variably located at a certain distance from the mitral annulus (frequently increased in case of severe MR with annular dilation),^30^ and circumflex coronary artery compression has been frequently observed.^31^–^33^

**Figure 3:**
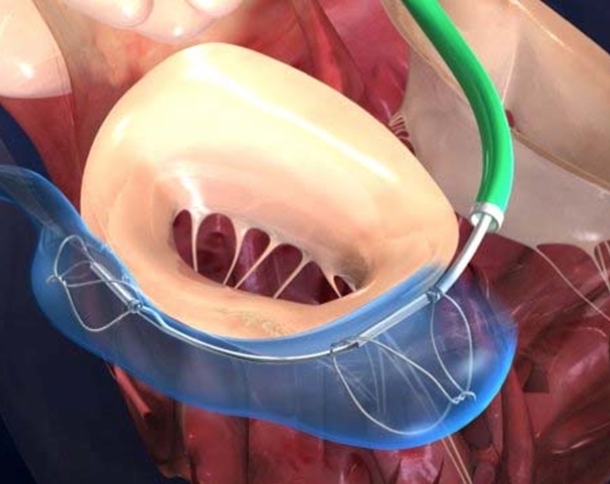
**Coronary Sinus Annuloplasty: the CARILLON Concept.** The Carillon device is composed of a nitinol bar with two anchors. The device is implanted in the coronary sinus to foreshorten the adjacent mitral annulus.

The Cardiac Dimension *CARILLON* (Cardiac Dimension, Kirkland, USA) is composed of two nitinol anchors (distal anchor placed in the great cardiac vein (GCV) and proximal anchor in the proximal CS) linked by a bridge element (Figure 3). Initial results have been promising,^34^ and it is now available for commercial implantation in Europe within a prospective post-market registry (PRIME) on-going to assess long-term safety and efficacy in up to 300 patients.

A previously investigated device was the Edwards *Monarc* (Edwards Lifesciences, Irvine, USA)^33^ that consists in a distal anchor placed at the transition between the anterior interventricular vein and the GCV, a proximal anchor placed in the ostial CS, and a spring-like bridge connecting the two. Further evaluation was suspended due to the slow enrollment in the dedicated clinical trial, but more importantly due to an excessive (and unexpected) rate of complications, including coronary occlusions. The Viacor *PTMA* (Viacor, Wilmington, USA)^35^ was made up of a plastic CS catheter containing up to three nitinol rods to provide incremental cinching/pushing of the posterior annulus. PTMA investigations have been suspended due to a series of device-related adverse events, including circumflex artery occlusion and fatal CS perforation.^31^,^36^,^37^

A more aggressive concept has been developed by the National Institute of Health. The *Mitral Cerclage* (NIH, Rockville, USA) creates a loop around the mitral annulus and left ventricular outflow tract (LVOT), entering through the CS ostium, passing through the anterior interventricular vein and returning near the CS ostium, perforating the myocardium either coming out in the right ventricle and passing through the anterior tricuspid commissure, or directly coming out through the septum in the right atrium; the loop is then tightened and secured near the CS ostium.^38^ Differently from the other coronary sinus devices, the Mitral Cerclage offers the opportunity of circumferential remodeling of the mitral annulus, using the coronary sinus as a support.

### Cinching Devices

These technologies force septo-lateral annular reduction through the approximation of two devices connected by a bridge. The reduction of this dimension is expected to be particularly important for MR reduction and in the functional setting.^39^

The *Ample PS3* System (Ample Medical, Inc., Foster City, CA, USA) consists of a CS anchor (“T bar”) and an interatrial septal anchor at the level of the fossa ovalis linked by an adjustable bridge; the device is designed for specific septal-lateral reduction at the P2 level. Clinical experience is limited to a temporary implant in two patients, in whom the device appeared safe and effective.^40^

The Myocor *i-Coapsys* (Edwards Lifesciences, Inc., Irvine, CA) is the percutaneous version of the surgical Coapsys, a surgical device to reshape the left ventricle. Large-scale data from the surgical RESTOR-MV trial suggest that, besides the MR reduction, the Coapsys can produce a significant LV restoration effect,^41^ also reducing myocardial fiber stress.^42^ The interventional device consists of two epicardial pads (anterior and posterior) connected by a load-bearing transventricular chord, all deliverable through a port inserted in the pericardium, with a percutaneous sub-xyphoid approach. Feasibility and safety of the i-Coapsys has been demonstrated in preclinical animal setting,^43^ and human initial experience has also been reported (Pedersen W. Failure Analysis for Percutaneous MV Repair Devices, TCT Meeting, San Francisco 2009).

The Mardil *BACE* (Basal Annuloplasty of the Cardia Externally; Mardil, Inc., Morrisville, NC) is a wide band with an inflatable chamber that is slipped externally around the base of the beating heart without cardiopulmonary bypass. The chamber can be inflated by saline through subcutaneous ports, and their volume can be adjusted intra- and postoperatively, thus remodeling the mitral valve annulus and sub-valvular apparatus. The surgical device seems effective in functional MR reduction,^44^ and a percutaneous development has been announced.

### Direct Annuloplasty

The implantation of devices directly into the mitral annulus more closely reproduces surgical annuloplasty. Only the posterior annulus is usually targeted, since the anterior annulus remains a more challenging structure due to the close vicinity of the aortic valve. Annular calcification, circumflex artery, and the potential for leaflet damage remain of concern for direct annuloplasty approach.

The *Accucinch* System (Guided Delivery System, Santa Clara, USA) is designed to implant a series of anchor elements under the posterior mitral annulus, in the sub-valvular space, from trigone to trigone. All anchors are connected by a cable that is used to cinch the annulus and the basal portion of the left ventricle. The Accucinch is delivered through a retrograde transfemoral route. The feasibility and the safety of the device have been shown in 10 patients, with no conversion to surgery and no 30-day major events; however, mitral regurgitation reduction was inconsistent, most probably due to the challenge of implanting the anchors from trigone to trigone and close enough to the annulus (Kleber F. GDS Accucinch Program Update, TCT Meeting, Miami 2012).

The *Mitralign* device (Mitralign, Tewksbury, USA) also uses a transfemoral retrograde approach to deliver pairs of pledgets connected with a suture. Each pledget pair can be cinched to achieve selective plications of the annulus. More than two pairs of pledgets can be implanted along the posterior annulus. A prospective, single arm feasibility and safety study is on-going to obtain CE mark. Available data reported that 36 patients have been enrolled, and 24 treated; 5 of them have reached 1 year follow-up. Although final results are not yet available, in the first 15 patients no procedural death and one pericardial tamponade occurred. After 1 year an average reduction of one grade of MR was observed along with minimal reduction in LV dimensions (Grube E. Mitralign Direct Annuloplasty, TVT Meeting, Vancouver 2011; and Buellesfeld L. Mitralign Program Update, TCT Meeting, Miami 2012).

**Figure 4:**
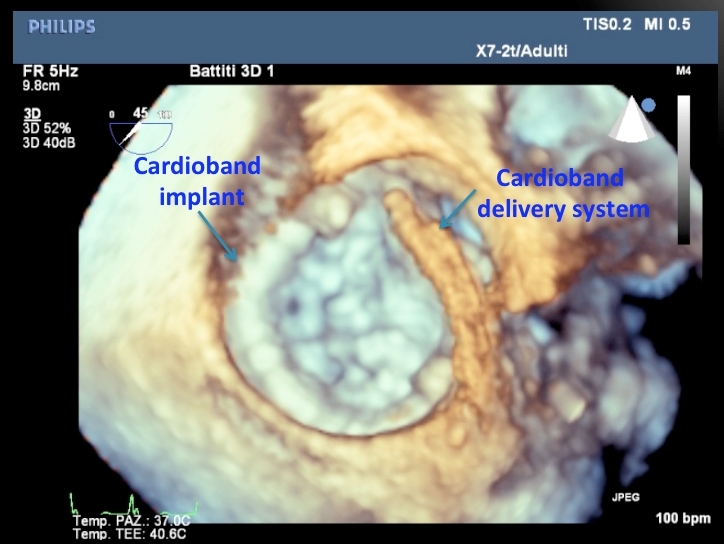
**The Direct Annuloplasty Cardioband System in a Patient Immediately after Implantation.** The device closely reproduces a surgical annuloplasty band, but is implanted percutaneously, with a transseptal approach.

The *Cardioband* System (Valtech Cardio Ltd, Or-Yehuda, Israel) is a transcatheter-implantable surgical-like ring (Figure 4). Differently from the previous devices, and similar to the MitraClip, it is delivered anterogradely via the right femoral vein and through a transseptal puncture. The Cardioband is a polyester prosthetic tube (band) sequentially fixed by helical anchors, from the antero-lateral to the postero-medial trigone; after implantation the band is shortened under echo-guidance on the beating heart, advancing a size-adjusting tool over a connecting cable. Results in 15 swine using a transatrial access have been promising,^45^ and an initial first-in-man experience has been safely and successfully started.

### Energy-mediated Annuloplasty

These technologies can be considered a subgroup of direct annuloplasty devices. However, rather than obtaining annular remodeling by implanting a device, they aim to reduce annular length by collagen shrinking through delivering of different energies. Safety remains a concern in terms of damage to surrounding structures (leaflets, myocardium, coronary sinus, circumflex artery, aortic valve) and thrombus formation.

The *QuantumCor* (QuantumCor, San Clemente, CA, USA)is composed of a malleable radiofrequency probe with seven electrodes. Available preclinical data on sheep showed significant antero-posterior annular reduction together with some degree of MR decrease and no sign of damage of surrounding structures.^46^

The *ReCor* (ReCor Medical, Ronkonkoma, NY, USA) device is a balloon catheter with a cylindrical piezoelectric ceramic transducer located towards the distal end, introduced in the left atrium through transseptal approach. The transducer converts electrical energy to acoustic energy, which is then delivered radially through a balloon inflated with a contrast-saline mixture at the mitral valve annulus site, without contact. In a series of 33 dogs, one death due to energy-induced ventricular fibrillation occurred and modest annular diameters reduction was observed, although no nearby structure was damaged.^47^

## PARAVALVULAR LEAK CLOSURE

Paravalvular leakage is a complication of prosthetic valve implantation occurring in 5%–15% of cases. Surgical re-operation is associated with higher morbidity and mortality and does not always guarantee a definite solution since prostheses detachment recurrence is not infrequent.

**Figure 5:**
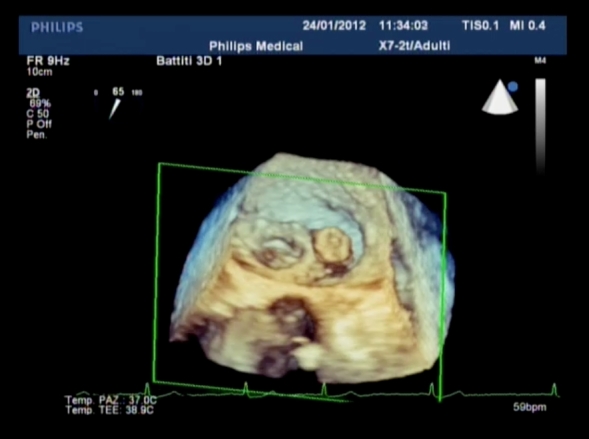
**Perivalvular Leak Closure with the Amplatzer AVPIII Device.** This 3D image shows a vascular plug implanted in a lateral leak of the mitral valve. The role of echo imaging is of paramount importance in this field.

Although it remains more challenging than aortic leak closures, in recent years transcatheter mitral leak closure had emerged as a new valid option. Procedural success rate has improved over the years, ranging from 60% to 90% in the different series, with different kinds of Amplatzer occluders (vascular plugs, atrial septal defect, patent ductus arteriosus).^48^ Imaging is fundamental for preoperative patient assessment and intra-procedural guidance. Accurate planning relies on 3D transesophageal echocardiography and coronary CT-scan with multiplanar, 3D, and 4D reconstructions. Shape, size, and location of the defect must precisely be evaluated, as well as relationships between heart structures and chambers and between the heart itself and the thoracic wall. Intra-operative guidance is performed by both 3D echocardiography and fluoroscopy (Figure 5). Mitral leak closure can be accomplished both through a venous transfemoral transseptal route and by a transapical access.^49^

## MITRAL VALVE IMPLANTATION

Although repair is currently the leading method to treat mitral valve regurgitation in surgical practice, replacement is associated with a number of potential advantages, particularly suitable for a transcatheter approach: the procedure can be more reproducible; it can be applied to the majority of patients, and may provide more predictable results. However, mitral valve anatomy brings unique and complex features that make transcatheter valve implantation much more challenging than in the aortic position. The mitral annulus is asymmetrical, non-tubular, and frequently not calcified, so that the main problem for any kind of mitral prosthesis remains anchoring, since radial force would not be effective and could cause serious complications. Left-ventricle outflow tract obstruction and aortic valve deformation (that could derive from a large and rigid mitral stent) are also major concerns. Moreover, leaks in the mitral position would be poorly tolerated, both hemodynamically and in terms of hemolysis because of the elevated pressure gradients. Mitral valve implantation is not yet routinely available in the clinical setting, but several devices are currently under development.

The *CardiAQ* (CardiAQ Valve Technologies, Inc., Winchester, MA, USA) prosthesis has been the first to reach human implantation in 2012, although only one case has been reported so far (Sondergaard L. Transcatheter Mitral Valve Implantation: CardiAQ, TCT Meeting, Miami 2012). It is transseptally delivered and self-anchoring without the need of radial force. The first patient showed early good implantation and hemodynamic result, but died after 3 days due to multi-organ failure. Autopsy did not reveal any prosthesis issue.

The *Lutter prosthesis* (Tendyne Medical, Inc., Baltimore, MD, US) has been successfully implanted transapically in numerous porcine models. The latest version of the prosthesis is made of a flat ring (atrial fixation system) connected at a 45° angle to the tubular stent that accommodates a 28-mm trileaflet bovine pericardial valve; between the base of the stent and the apex, neo-chordae act as ventricular fixation system. A waterproof membrane is sutured in the atrial ring and over the ventricular component to guarantee sealing, minimize paravalvular leakage, and allow easier repositioning. In the latest animal series the prosthesis was implanted with optimal results and without any complications in 5/6 animals.^50^

The *Tiara* (Neovasc, Inc., Richmond, British Columbia, Canada) is a transapical self-expandable valve; its atrial portion is designed specifically to fit the saddle-shaped mitral annulus: the D-shape matches the natural shape of the mitral orifice and prevents impingement of the left ventricular outflow tract (LVOT). The ventricular portion of the device comprises a covered skirt to prevent paravalvular leak and three anchoring structures to capture the fibrous trigones of the posterior mitral leaflet. First, the atrial portion is deployed and oriented; the valve is then pulled downward to seat the atrial flange firmly on the floor of the atrium, and the three ventricular anchor structures are deployed. Finally the ventricular skirt and valve leaflets are released from the catheter, allowing the device to begin functioning. In all stages until the final step of ventricular deployment, the valve is retrievable and repositionable. Tiara valves were implanted with early successful results in 29/36 domestic swine.^51^ Precise data and longer follow-up are needed to evaluate correctly this new transcatheter heart valve, but the initial report seems promising. The D-shape in particular appears a clever idea both to protect the aortic valve/LVOT and to optimize contact and interaction between the atrium and the mitral prosthesis.

The *Endovalve-Herrmann prosthesis* (Endovalve, Inc., Princeton, NJ, USA) is implanted from the left atrium via a right mini-thoracotomy on a beating heart. The device is a foldable nitinol structure that attaches to the native valve with specially designed grippers, is fully valve sparing, and repositionable before release. Animal modelshave been successful, and a true percutaneous version is planned.

**Figure 6:**
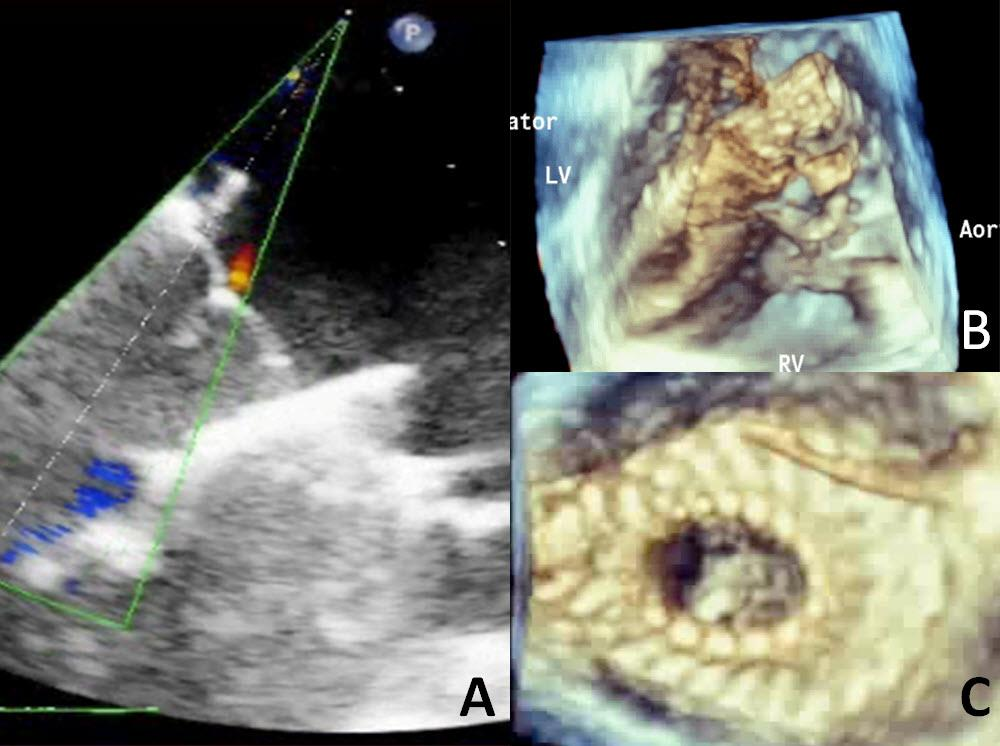
**The Cardiovalve (Valtech-cardio).** **A:** The Cardiovalve *in situ* in an animal model, with no perivalvular regurgitation and optimal competence of the valve. **B:** A 3D view of deployment. **C:** A 3D view from the atrial perspective of the implanted Cardiovalve.

The *CardioValve* (Valtech Cardio Ltd, Or-Yehuda, Israel) is also currently delivered off-pump through the left atrium and is currently in preclinical animal development(Figure 6).

### Valve-in-Valve/Valve-in-Ring

**Figure 7:**
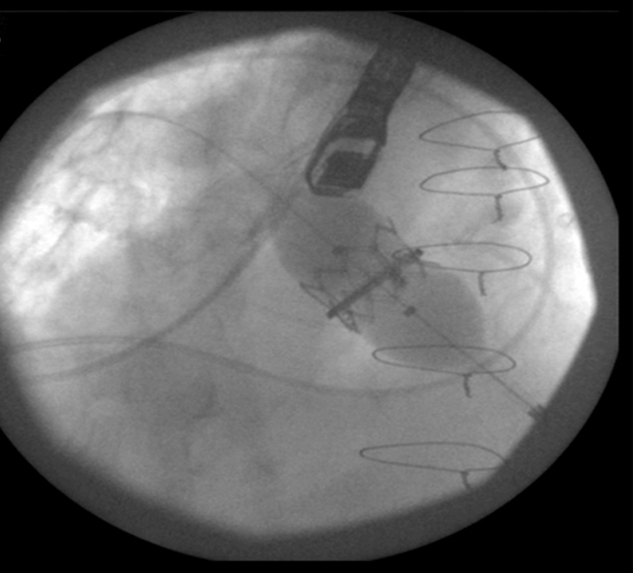
**Valve-in-ring Procedure.** Fluoroscopic image of Edwards SAPIEN XT 26 deployment into a previously implanted C-E Physio ring 28.

Although transcatheter prosthesis implantation on the native mitral valve is not yet available for clinical use, valve in valve (ViV) and valve in ring (ViR) procedures are performed routinely (on an off-label basis). The previously implanted bioprosthesis or ring provides an ideal support for the successful implantation of the currently available transcatheter aortic valve prostheses, which commonly use radial force (Figure 7). Most procedures have been performed with the Edwards SAPIEN (Edwards Lifesciences, Inc., Irvine, CA) valve.

Data from the Global Registry recently (Dvir D. Update from the Global Valve-in-Valve Registry, TCT Meeting, Miami 2012) reported a 30-day and 1-year mortality of 12% and 25%, respectively, and an impressive improvement in symptoms early after the procedure. The most used access has been the transapical (85%), but transatrial, transjugular, and transfemoral approaches have also been performed.

## CONCLUSIONS

Transcatheter mitral valve repair with the MitraClip System has proved excellent safety results and good efficacy results in high-risk patients and is already a real alternative to surgery in such a population. Furthermore, MitraClip is evolving as a device therapy for congestive heart failure, targeting patients who were not referred for surgery in the past.

To expand further the field of transcatheter mitral valve treatment, several devices, addressing many different anatomical and pathophysiological concepts (from annuloplasty to valve implantation), are under development.

Transcatheter mitral techniques should be considered the natural and inevitable evolution of mitral surgery. In the context of such a wide spectrum of different available therapies, the patient-centered care and the heart-team approaches are fundamental to provide effective and individualized treatments.

## Abbreviations

CABG: coronary artery bypass surgery;
CS: coronary sinus;
DMR: degenerative mitral valve regurgitation;
FMR: functional mitral valve regurgitation;
GCV: great cardiac vein;
LV: left ventricle;
LVOT: left ventricular outflow tract;
MR: mitral valve regurgitation;
MV: mitral valve;
NYHA: New York Heart Association.
